# *STRN-ALK* Rearranged Malignant Peritoneal Mesothelioma With Dramatic Response Following Ceritinib Treatment

**DOI:** 10.1200/PO.19.00048

**Published:** 2019-07-11

**Authors:** Jan H. Rüschoff, Elise Gradhand, Abdullah Kahraman, Helen Rees, Jane L. Ferguson, Alessandra Curioni-Fontecedro, Martin Zoche, Holger Moch, Bart Vrugt

**Affiliations:** ^1^ University Hospital Zurich, Zurich, Switzerland; ^2^ Dr Senckenberg Institute of Pathology, University Hospital Frankfurt, Germany; ^3^ Bristol Royal Hospital for Children, Bristol, United Kingdom; ^4^ Royal Devon and Exeter National Health Service Foundation Trust, Exeter, United Kingdom

## INTRODUCTION

Malignant mesothelioma (MM) is a rare neoplasm arising from the mesothelial cell lining of the pleura (80% to 90%), but it can also develop in the peritoneum (10% to 15%), pericardium, and tunica vaginalis.^1,2^ Because of the long latency of the disease, the age at diagnosis ranges from 54 to 65 years^3-5^; thus, MM rarely occurs in young patients and children. The frequency of both pleural and peritoneal MM is almost equal in patients younger than age 40 years. In addition, previous asbestos exposure is less common in peritoneal compared with pleural MM.^6^ Peritoneal mesothelioma commonly presents with vague and unspecific symptoms, including abdominal distension and weight loss.^7^ Consequently, the diagnosis of peritoneal mesothelioma generally is made at an advanced stage of the disease,^8^ which partly explains the poor clinical outcome, even after optimal oncologic and surgical treatment. The median survival is less than 12 months in the majority of patients.^9,10^ Patients with MM are not routinely analyzed for the presence of potentially drug-targetable gene mutations, including anaplastic lymphoma tyrosine kinase (*ALK*). By using targeted next-generation sequencing of tumor DNA and RNA, Hung et al^11^ recently identified *ALK* rearrangements with three novel *ALK* fusion partners in a small subgroup of patients with peritoneal MM. Fusion partners were *ATG16L1*, *STRN*, and *TPM1*. *ATG16L1-ALK, STRN-ALK*, and *TPM1-ALK* fusions were all in-frame alterations. The *ALK* breakpoint was mapped to intron 19 in all three cases, the *STRN* breakpoint to intron 3, the *ATG16L1* breakpoint to intron 2, and the *TPMI1* breakpoint to the beginning of exon 9.

Here we report a 13-year-old girl with peritoneal mesothelioma harboring an *STRN-ALK* gene fusion, which was identified by comprehensive genomic profiling (CGP). The patient demonstrated a dramatic response with marked clinical improvement after treatment with ceritinib.

## CASE REPORT

**FIG 1.**
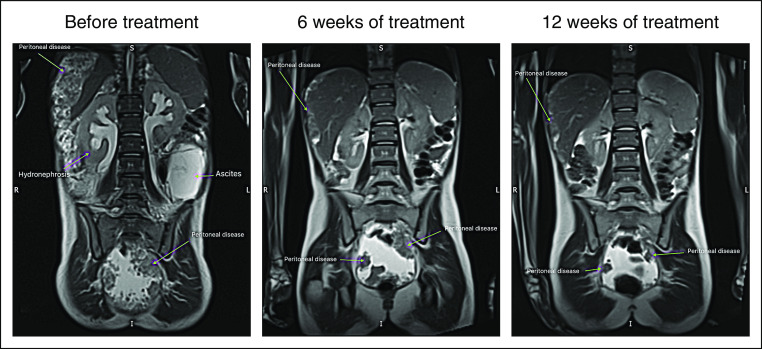
Gadolinium-enhanced magnetic resonance imaging scans of the abdomen before and at 6 weeks and 12 weeks after starting ceritinib. Images before treatment demonstrate large-volume ascites, bilateral hydronephrosis, and widespread peritoneal soft tissue deposits particularly within the pelvis (all findings marked by green arrows). Follow-up imaging 1 month after treatment was started showed a significant reduction in tumor bulk in these areas and reduction in volume of ascites. Hydronephrosis has resolved. At 12 weeks after treatment, there is a further reduction in the volume of peritoneal disease and ascites.

A 13-year-old previously healthy girl presented to her general physician with a 4-month history of increasing abdominal girth. She felt otherwise well, although her appetite had reduced slightly, and she was attending school as usual and continuing her sports activities. On admission to the hospital, significant ascites was noted, and a magnetic resonance imaging scan demonstrated extensive peritoneal disease. Images before treatment showed large-volume ascites, bilateral hydronephrosis, and widespread peritoneal soft tissue deposits particularly within the pelvis and around the liver with scalloping of the liver margin (Fig 1). She had no history of asbestos or radiation exposure. Peritoneal biopsies were obtained by an explorative laparoscopy and submitted for a histologic and molecular work-up.

## PATHOLOGY

**FIG 2.**
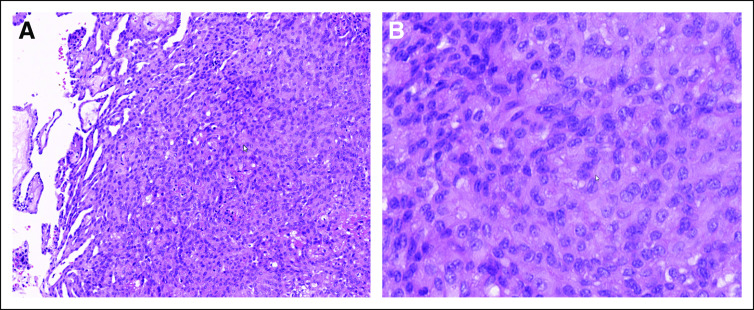
(A) Peritoneal biopsy with a neoplasm showing a tubulopapillary and solid growth pattern (original magnification ×5). (B) Higher magnification (original magnification × 20) shows relatively bland tumor cells with slightly irregular nuclei and inconspicuous nucleoli. Hematoxylin and eosin stain was used for (A) and (B).

Multiple peritoneal biopsies revealed a tumor with a mixed tubulopapillary and solid growth pattern. Immunohistochemistry with positivity for CK5/6 and calretinin in the absence of expression of the epithelial marker BerEp-4 and claudin-4 confirmed the mesothelial origin of the tumor. Even though necrosis and mitotic activity (less than 1 mitosis per 10 high-power fields) were not evident, growth pattern, cytonuclear atypia of the tumor cells, and immune profile fulfilled the criteria for malignant epithelioid mesothelioma (Figs 2A and 2B). Immunohistochemistry showed retained expression of BAP-1, p16, and MTAP protein, whereas a *CDKN2A* fluorescent in situ hybridization (FISH) analysis revealed no loss of the 9p21 chromosome.

## COMPREHENSIVE GENOMIC PROFILING

**FIG 3.**
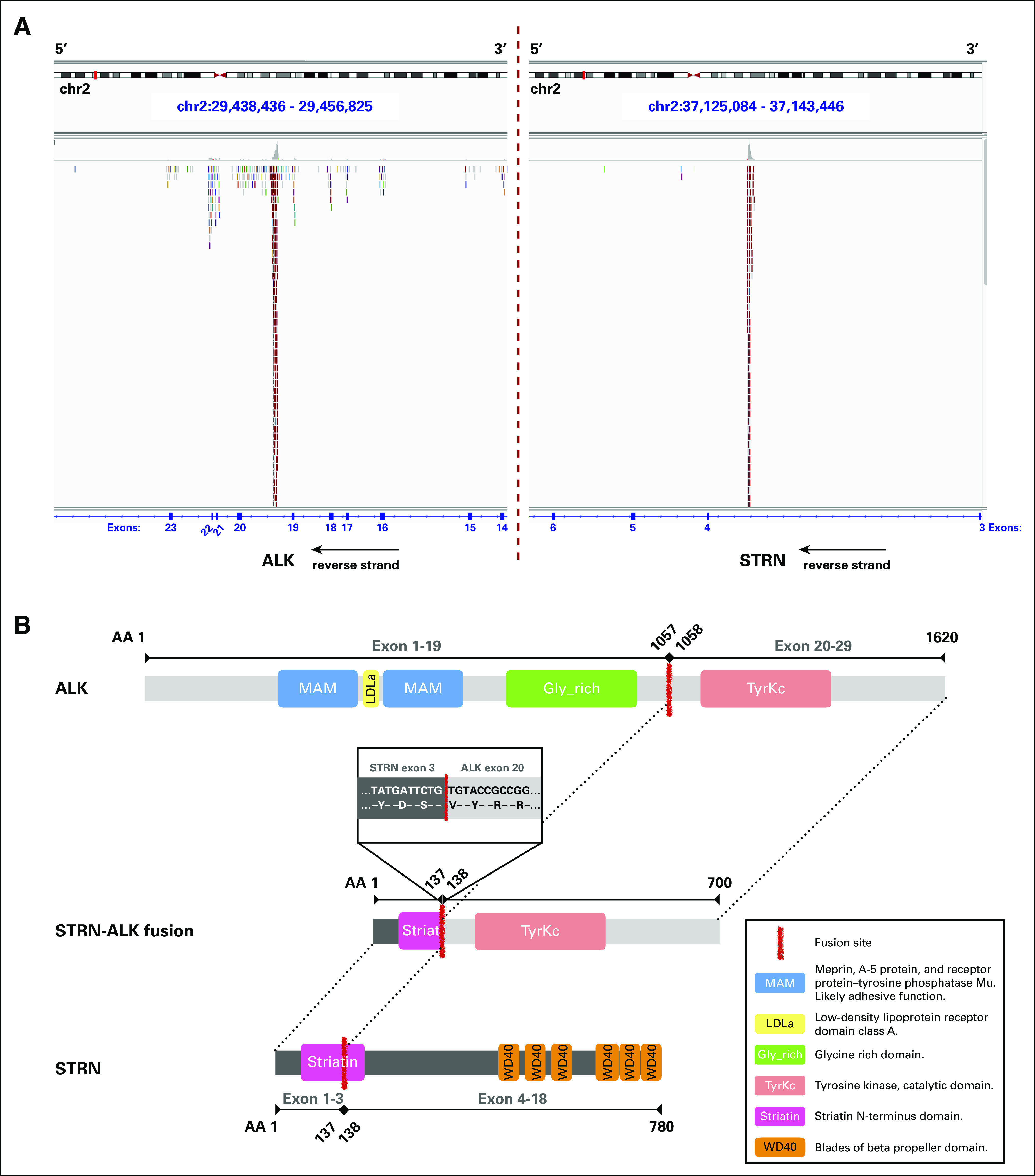
Next-generation sequencing data showing the *STRN-ALK* fusion. (A) Alignment of 176 paired-end reads spanning both breakpoints in *STRN* and *ALK* are shown on the human reference genome (hg19). Position of depicted genomic regions are labeled in blue at the top. The breakpoints in *STRN* and *ALK* were found in intron 3 (chr2:37,134,246-37,134,536) and intron 19 (chr2:29,447,483-29,447,805), respectively. (B) Schematic representation of the *STRN-ALK* fusion showing the 700-amino-acid (AA)-long fusion gene, with 137 AAs originating from the N terminus of *STRN* and the remaining 562 AAs originating from the C terminus of *ALK*. Although the Striatin domain in the *STRN-ALK* fusion is likely nonfunctional because of its premature truncation at its C-terminal region, the tyrosine kinase domain of *ALK* remains intact.

**FIG 4.**
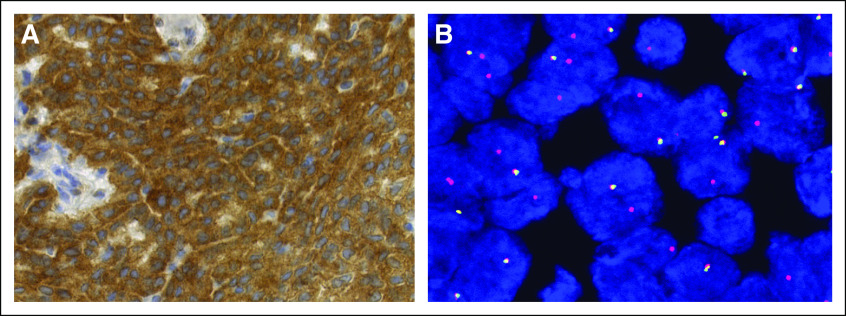
(A) Immunohistochemical staining showing strong expression of ALK protein (original magnification ×40). (B) *ALK* fluorescent in situ hybridization break-apart probes showing one fused red-green signal and one red signal in 96% of the tumor cells compatible with 5′ *ALK* deletion (original magnification ×100).

To identify drug-targetable mutations, CGP using next-generation sequencing using FoundationOne (Foundation Medicine, Cambridge, MA) was performed on the exons of 315 cancer-associated genes.^12^ In addition, intronic regions of 28 genes that are known to be involved in somatic rearrangements were sequenced, and the microsatellite instability and tumor mutational burden were assessed.^13^ The sequencing analysis identified an isolated *STRN-ALK* rearrangement and three variants of unknown significance, namely *FGFR2* (c.1562A>G, p.D521G), *KMT2D* (c.5077C>T, p.R1693W), and *PRKDC* (c.6206G>A, p.R2069Q). Mutational burden was low (three mutations per megabase pair), and microsatellites were found to be stable. Genetic alterations in the *NF2* gene were not detected nor were additional oncogenic drivers known to be present in malignant mesotheliomas (Fig 3). Subsequently, the *STRN-ALK* rearrangement was validated by immunohistochemistry showing strong protein expression of ALK (clone 5A4; Fig 4A). A FISH analysis revealed a 5′ *ALK* deletion in 96% of the tumor cells (Fig 4B).

## TREATMENT AND FOLLOW-UP

After the histologic diagnosis was established, the patient was initially treated with four cycles of cisplatin and pemetrexed without clinical or radiologic response. She was reviewed by a national peritoneal mesothelioma surgical team who staged her laparoscopically and deemed her case not amenable to cytoreductive surgery and hyperthermic intraperitoneal chemotherapy. Therefore, palliative care with ascites drainage (6 L/week) was provided. Molecular testing by CGP was attempted to obtain possible options for targeted treatment. After the *STRN-ALK* rearrangement was detected, treatment was started with off-label oral ceritinib 450 mg once per day. Follow-up imaging 6 and 12 weeks after the start of treatment demonstrated a significant reduction in the previously observed abdominal mass and resolution of ascites and hydronephrosis. At 3 months after treatment, there was a further reduction in the volume of peritoneal disease and ascites (Fig 1). Except for a transient and self-limiting rise in alanine aminotransferase (ALT), no adverse effects were noted.

## DISCUSSION

We report a dramatic tumor regression after the first cycle of ceritinib in a 13-year-old girl with *ALK*-rearranged peritoneal MM. The ALK receptor can be translocated, mutated, or overexpressed in non-squamous non–small-cell lung cancer, inflammatory myofibroblastic tumors (IMTs), and anaplastic large-cell lymphomas (ALCLs).^14^ It is well known that pediatric patients with IMTs or ALCL can show strong and sustained clinical response to the inhibitory effects of the ALK-targeting drug crizotinib.^15^ Peritoneal MM is an extremely rare tumor in children, but an almost complete response to an ALK-targeting drug has never been described before. Recently, Loharamtaweethong et al^16^ reported a 10-year-old girl with an *ALK*-translocated peritoneal MM detected by FISH and immunohistochemistry, but the exact fusion partner was not determined. This patient received combined treatment with cisplatin and gemcitabine, which resulted in stable disease but not remission. This patient strengthens the importance of access to specific drugs for rare tumors with rare molecular alterations. Rearrangements in the *ALK* gene have been identified in a small subset of patients with peritoneal MM and are thought to be involved in its pathogenesis. Peritoneal MM with *ALK* rearrangements most often occurs in female patients younger than age 40 years with no history of asbestos exposure or radiotherapy. Genetic alterations frequently seen in pleural and peritoneal mesothelioma, including *CDKN2A*, *BAP-1*, *NF2*, and *SETD2*, are not detected in patients with *ALK*-rearranged disease.^11^ Even though the prevalence of *ALK*-rearranged peritoneal mesotheliomas is low, *ALK* alterations seem to be restricted to peritoneal mesothelioma only and do not occur in pleural MM. By using *ALK* FISH, Salvi et al^17^ were unable to detect *ALK* translocations in 106 patients with pleural mesothelioma with different histologic subtypes (epithelioid [n = 60], sarcomatoid [n = 34], and biphasic [n = 12]). This is consistent with our observation, because we were unable to demonstrate ALK expression in a tissue microarray containing tumor samples from 221 patients with pleural mesothelioma, including 22 females of whom three were younger than age 40 years (unpublished data).

In our patient’s tumor sample, we identified a rare *ALK* fusion by CGP. The platform used in this study can detect all *ALK* alterations (including *ATG16L1* and *TPM1* fusions) and has been extensively validated.^12^ Even in *ALK* FISH-negative patients, *ALK* rearrangements could be identified by using this method.^18^ In our patient, we detected an *STRN-ALK* fusion with similar breakpoints but with a fusion protein identical to that in the *ALK*-rearranged patient described by Hung et al.^11^ The *STRN-ALK* fusion was an in-frame alteration with breakpoints for *STRN* in intron 3 and *ALK* in intron 19.

Patients with *ALK*-rearranged MM cannot be distinguished from their conventional counterparts, but the differences in clinical presentation (young patients with no history of asbestos exposure) and the detection of isolated *ALK* mutations would justify a separate tumor entity of *ALK*-rearranged mesothelioma. In the absence of genetic alterations of *CDKN2A*, *BAP-1*, *NF2*, and *SETD2*, it is tempting to speculate that fusions involving the *ALK* gene may represent the predominant oncogenic driver in peritoneal MM in young female patients. On the basis of the success of therapeutic ALK inhibition in our patient, *ALK* fusion screening by CGP is highly advised in peritoneal MMs, especially in young patients. The promising response in our patient also stresses the need for investigations involving a larger cohort of young female patients with *ALK*-rearranged peritoneal MM to determine the efficacy and durability of ALK inhibition. However, such a clinical trial is difficult to perform, given the rarity of this MM subtype. Alternative approaches for obtaining the data needed regarding response to ALK inhibitor therapy in this rare entity could be a search within international cancer registries, such as the European Platform on Rare Disease Registration, or inclusion of *ALK*-rearranged peritoneal MM into basket trials for variable ALK-rearranged neoplasia (eg, lung cancer, anaplastic large-cell lymphoma, and inflammatory myofibroblastic tumors). Positive response to ALK inhibition in a larger cohort of patients with peritoneal mesotheliomas would justify a separate entity of *ALK*-rearranged MM in a future WHO classification.
